# Bone Remodeling by Rapid Maxillary Expansion (RME): Evidence from the Monitoring of Bone Turnover Biomarkers in Salivary Matrix

**DOI:** 10.3390/dj14070444

**Published:** 2026-07-16

**Authors:** Vincenzo Brescia, Domenico Ciavarella, Roberto Lovero, Maria Bisceglia, Mauro Lorusso, Francesco Testa, Lucia Varraso, Antonietta Fontana, Francesca Di Serio, Vito Crincoli, Angela Pia Cazzolla

**Affiliations:** 1 Clinical Pathology Unit, AOU Policlinico Consorziale di Bari-Ospedale Giovanni XXIII, 70124 Bari, Italy; vincenzo.brescia@policlinico.ba.it (V.B.); lucia.varraso@policlinico.ba.it (L.V.); antonietta.fontana@policlinico.ba.it (A.F.); francesca.diserio@policlinico.ba.it (F.D.S.); 2 Department of Clinical and Experimental Medicine, Dental School of Foggia, University of Foggia, 71122 Foggia, Italy; domenico.ciavarella@unifg.it (D.C.); maria.bisceglia@unifg.it (M.B.); mauro.lorusso@unifg.it (M.L.); francesco.testa@me.com (F.T.); 3 Interdisciplinary Department of Medicine, University of Bari “Aldo Moro”, Piazza G. Cesare 11, 70124 Bari, Italy; vito.crincoli@uniba.it; 4 Department of Medicine and Surgery, LUM University, 70010 Bari, Italy

**Keywords:** rapid maxillary expansion, bone turnover markers, orthodontics interceptive

## Abstract

**Background/Objectives:** Orthodontic treatment with a Rapid Maxillary Expander (RME) induces stress on the mid-palatal suture and involves the surrounding craniofacial sutures, leading to significant variations in bone turnover markers (BTMs). The aim of this study was to evaluate whether monitoring biomarkers of bone resorption and deposition in saliva could provide indications for assessing RME treatment effectiveness in pediatric patients. **Materials and Methods:** The study was conducted at the Dental Clinic of Foggia in collaboration with the Clinical Pathology Unit of the Policlinico-Bari from January 2023 to September 2025. Salivary samples were collected using cotton swabs (SALIVETTE^®^, SARSTEDT, Nümbrecht-Elsenroth, Germany) from 47 patients (aged 9–13 years) presenting with Class I dental and skeletal relationships and transverse maxillary deficiency. Samples were collected at five time points: before RME application (t0), and 15 (t1), 30 (t2), 45 (t3), and 60 (t4) days after the start of expansion. For pairwise comparisons of concentrations at different time points the Wilcoxon signed-rank test was used. Analysis of Variance (ANOVA) and Ordinary Least Squares (OLS) multiple regression were used to analyze the relationships between different biomarker concentrations over time. Spearman correlation was used to assess relationships between BTM concentrations at the different time points. **Results:** The Wilcoxon test showed that the medians of the differences for P1NP measurements were statistically significant at all time points, except between t0 and t1. Differences in PTHrP concentrations were significant, except for the comparison of t2 and t3 relative to t4. No statistically significant differences were observed for TRAcP across any measurement times. ANOVA yielded a high F-value for P1NP (F = 3.6128; *p* = 0.0128), indicating significant variation, whereas the F-ratios for PTHrP (F = 1.3329; *p* = 0.2736) and TRAcP (F = 1.3915; *p* = 0.2534) were close to 1, suggesting non-significant variability. Spearman correlation indicated that P1NP showed the strongest relationships with temporal variables. **Discussion and Conclusions:** Rapid maxillary expansion results in a significant increase in P1NP levels, reflecting collagen deposition and the onset of bone formation. This preliminary study on saliva highlights how biochemical changes can support clinicians in monitoring and validating the effectiveness of treatment.

## 1. Introduction

Rapid maxillary expansion (RME) is the orthopedic skeletal expansion of the maxilla, performed with a device that has a skeletal effect and is anchored to the teeth [1,2].

This procedure is commonly used to treat transverse maxillary deficiencies, characterized by abnormally low maxillary growth. Transverse deficiency of the upper jaw recognizes a multifactorial etiopathogenesis [2,3], such as primary congenital alterations, trauma, infections, metabolic disorders, mouth breathing, atypical swallowing, and thumb or pacifier sucking [3,4,5,6].

Maxillary transverse deficiency manifests clinically as an abnormal relationship in the transverse plane between the maxillary arch and the mandibular arch of skeletal origin and it is frequently associated with dental crowding, crossbite, Class II and III malocclusion, and sometimes with temporomandibular joint dysfunction [7,8,9]. Regarding dental crossbite, it can be unilateral or bilateral, and it can involve one or more teeth [8,9].

Many types of appliances have been developed to proceed with maxillary expansion with different expansion speeds or different application timings: the Rapid Maxillary Expander (RME), the Leaf Expander^®^ (Slow and Constant Expansion), the Quad-Helix, screw expanders activated with lower frequency (once a week), or miniscrew-assisted expanders (MARPEs) [9,10]. The application of an RME during the developmental age involves the use of orthopedic forces for a limited period of time. These forces act mainly on the mid-palatal suture, with the aim of preserving the buccal bone of the roots of the first permanent molars [10,11]. The alveolar bone is protected thanks to a process of hyalinization that contrasts bone resorption, prevents dental displacement, and produces expansion at the level of the midpalatal suture [11]. Different activation protocols for these appliances lead to the opening of the palatal suture and an increase in palatal width [12,13].

An important aspect regarding RME is the timing of treatment, which plays a key role. In fact, the correction of the transverse deficiency of the maxillary bone must be performed in the early stages of maxillary development until the midpalatal suture (MPS) remains fibrous and poorly interdigitated. The maturation of the midpalatal suture is a dynamic process [1,2].

Melsen, through autopsy studies, identified three stages of development that progress from childhood to adolescence and are morphologically characterized by the progressive acquisition of a tortuous appearance, followed by significant interdigitation, and the formation of bone bridges until complete synostosis [14,15,16]. From a histological point of view, the midpalatal suture (MPS) is initially composed of dense, highly cellular, and vascularized connective tissue, which allows for a certain degree of mobility and orthopedic adaptation at an early age [1,16]. As skeletal maturation progresses, this structure undergoes a process of progressive ossification, in which sutural interdigitation increases, vascularization decreases, and finally, a decrease in cellularity and osteogenic activity occurs, with complete obliteration of the sutural space. This final phase of maturation is a true bone fusion (synostosis), which significantly limits the possibility of non-surgical orthopedic expansion of the maxilla [3,16,17].

The timing and methods of mineralization show enormous variability in the closure index due to diversity in skeletal maturation and different extrinsic functional demands of the specific sutural area. This process is not synchronous with chronological age and presents high inter-individual variability, influenced by biomechanical, genetic, and functional factors. For example, it has been demonstrated that the loss of functional load associated with edentulism induces substantial morphological alterations in the MPS, such as an increase in obliteration, a reduction in vascular lumen, and a significant reduction in sutural width, suggesting that functional occlusal stimulation plays a key role in maintaining sutural anatomical integrity [2,18].

Skeletal maxillary expansion is characterized by the application of a set force that acts by dividing the horizontal laminae of the palatine bone and the palatine processes. There is an individual response to rapid expansion that is determined by the patient’s sutural activity and depends on the patient’s stage of sutural maturation. Therefore, stimulation of sutural growth is possible only in individuals who have not reached the pubertal growth peak and have a diastasis, while in adult patients, expansion can only occur through numerous microfractures of the sutural region [19,20].

Stimulating growth and development at an early age or during the growth peak favors correction, unlike in adult patients without residual growth. Most authors argue that the suture begins to obliterate around 14–16 years and appears closed only in 5% of subjects aged 25. The average age beyond which orthopedic expansion is less likely to be obtained is 13–14 years in females and 15–16 years in males [6,21].

The mechanical load induced by RME is a powerful inducer of bone remodeling; the success of RME depends on bone remodeling with processes of bone resorption and deposition [22,23]. Various models have been developed to explain the orthodontic movement of teeth during RME and for monitoring the state of the bone while cellular and molecular events related to RME have been evaluated in only a few studies [16]. An increase in the number of osteoblasts around the palatal bones has been reported, suggesting active bone formation after 7 and 14 days [16,17,18,19]; in periosteal cells distributed along the medial surface of the bone and newly formed cartilage in the midpalatal suture an increase in the expression of alkaline phosphatase and type I collagen mRNA has been highlighted [24,25]. Studies have observed an increased layer of osteocalcin-positive osteoblasts covering the surface of newly formed bone following the mechanical load of RME [24,25,26]. RME results in an increase in the distance of the maxillary bones in the midpalatal suture and, consequently, an increase in the number of osteoblasts and the release of bone remodeling biomarkers also occurs. The efficacy of RME is correlated with the increase in the distance of the maxillary bones in the midpalatal suture, the increased number of osteoblasts, and the production of the main markers of bone remodeling [21,22,27].

Previous investigations on the biochemical response to RME have primarily focused on biomarkers measured in gingival crevicular fluid (GCF), including alkaline phosphatase, osteocalcin, and pro-inflammatory cytokines, which reflect local periodontal remodeling. However, no studies have evaluated bone turnover markers such as P1NP, PTHrP, or TRAcP in the salivary matrix during RME. Unlike GCF, saliva provides a non-invasive and easily repeatable medium that reflects broader craniofacial metabolic activity. To our knowledge, this is the first study to longitudinally monitor these specific biomarkers in saliva across multiple time points during RME, thereby offering a novel biochemical perspective on skeletal remodeling induced by maxillary expansion [28].

Orthodontic treatment with RME involves stress at the level of the palatal suture of the maxilla that could determine significant changes in the concentrations of salivary biomarkers of bone metabolism in a manner analogous to what occurs with fixed orthodontic treatment and mechanical load in OTM (Orthodontic Tooth Movement). The methodologies in use in clinical pathology laboratories have high analytical sensitivity and allow for the measurement of the most effective biomarkers of bone remodeling even at low concentrations. The evaluation of changes in the concentrations of bone turnover biomarkers in the salivary matrix could provide information on the biological phenomena occurring in the periodontal tissue during orthodontic treatment with RME. The aim of the present study was to evaluate whether the dosage of bone resorption and deposition biomarkers in the salivary matrix could provide indications for monitoring RME treatment in pediatric patients.

## 2. Materials and Methods

### 2.1. Study Design

The study was conducted at the Dental Clinic of Foggia from January 2022 to February–September 2025, in collaboration with the Clinical Pathology Unit of the Bari University Hospital. A total of 47 patients were recruited, all presenting with Class I dental and skeletal relationships, good gingival and periodontal health, aged between 9 and 13 years, and showing transverse deficiency of the maxilla. The study was approved by the Ethics Committees of the Bari University Hospital (Biomarkers of Bone Metabolism, Study No. 38359/COMET, 27 April 2021, BMOPed) and was carried out in accordance with the Declaration of Helsinki. All parents or legal guardians of the enrolled subjects signed informed consent for participation in the study.

### 2.2. Exclusion Criteria

Individuals with rare diseases causing alterations in calcium–phosphorus metabolism or oral manifestations and/or renal involvement, inflammatory bowel diseases, diabetes mellitus, or those who had received immunosuppressive drugs in the last three months were excluded. Furthermore, all subjects presenting with bleeding oral lesions of any nature, including mechanical ones, during any phase of sample collection were excluded. Patients with Bleeding on Probing (BOP) greater than 10%, Loe and Silness gingival index (GI) greater than 1, Loe and Silness plaque index (PI) above 0, and a Periodontal Probing Depth (PPD) greater than 3 mm were excluded. In addition, all patients who had undergone any type of previous orthodontic treatment in recent months, or who had not attended regular and periodic dental check-ups, were excluded.

### 2.3. Dental Evaluation

All study participants underwent professional session of supra- and subgingival scaling and also received repeated oral hygiene instructions during the period between one week and one month prior to saliva collection. Clinical parameters (BOP, PPD, GI, and PI) were measured for all teeth present in the dental arch; at each visit, PI, GI, BOP, and PPD were assessed. The evaluation of indices was performed on the control lower molars and on six sites for each tooth. Patients were instructed to maintain proper oral hygiene during all phases of their orthodontic treatment. The oral hygiene levels of participants were periodically assessed through their PI and GI scores. A PI score equal to 0 and a GI score below 1 are considered good indicators of oral health.

### 2.4. Orthodontic–Orthopedic Treatment

RME treatment involved RME appliance with solely dental anchorage. The appliance had a hyrax screw (palatal screw type S, Forestadent, Pforzheim, Germany, lift height 0.2 mm) and it was fixed with two bands on the permanent upper first molars using Transbond™ light-curing composite resin cement (3M Unitek, Monrovia, CA, USA) with occlusal rests on the first premolars or deciduous molars. The expansion screws used measured 9 or 12 mm, depending on the anatomy of the palate. Activation consisted of two turns per day for 15 days to ensure correction of the transverse relationships of the basal bones. After 15 days, the expander was locked and maintained in place for 9 months to achieve complete calcification of the mid-palatal suture.

### 2.5. Saliva Sample Collection Method

For hygienic collection of whole saliva using the passive salivation method, the Salivette^®^ (SARSTEDT, Nümbrecht-Elsenroth, Germany) system was employed. The procedure consisted of collecting saliva with a sterile synthetic fiber cotton roll, gently chewing the swab for two minutes, and transferring it into the designated container without manipulation. Samples were considered suitable if they showed no evidence of blood contamination; then, they were stored at a temperature between 4 and 8 °C and delivered to the laboratory within two hours of collection. Saliva samples were collected five times: before the start of orthodontic treatment (t0), at 15 days (t1), 30 days (t2), 45 days (t3), and 60 days (t4). All participants received written instructions to minimize potential errors during saliva collection. They were asked to fast for 2 h before sampling (excluding water) and to rinse the mouth with plain water, avoiding disinfectants or mouthwashes. All samples were collected under the direct supervision of healthcare personnel, who ensured the appropriateness of the procedure. Eligible specimens were stored at 4–8 °C for 24 h, centrifuged at 4000× *g* for 3 min, and subsequently kept at −30 °C until analysis.

### 2.6. Verification of Blood Contamination in Saliva Samples

The concentration of bone metabolism biomarkers in blood is higher than in saliva. Saliva samples contaminated with blood may affect dosage; therefore, all samples with Hgb values > 50 mg/dL, assessed using an automated spectrophotometric method (HIL) on the Alinity clinical chemistry analyzer (Siemens, Munich, Germany), were excluded.

### 2.7. Selection of Bone Metabolic Biomarkers (BTM)

The following were measured in the salivary matrix:

- Parathyroid hormone-related peptide (1–64) (PTHrP) which contributes to the regulation of bone development [29].
- Type I procollagen N-terminal propeptide (P1NP) [27,30] produced during the formation of the bone matrix by the cleavage of type I collagen and linked to osteoblastic activity.
- Tartrate-resistant acid phosphatase isoform 5b (TRAcP5b) [31,32] generated by osteoclasts, which participates in the degradation of type I collagen in the bone matrix and is useful in monitoring bone resorption processes.

### 2.8. Analysis of Bone Metabolic Biomarkers (BTMs)

The dosage of parathyroid hormone-related protein (1–64) (PTHrP) was measured using a competitive immunoenzymatic assay (ELISA) designed to detect the (1–34) subunit (Parathyroid Hormone-Related Protein (PTHrP) (1–34) EIA Kit, catalog no. EK-056-04, Phoenix Pharmaceuticals, Inc.; 330 Beach Rd., Burlingame, CA, USA) (measurement range 0.033–6000 ng/mL, limit of detection (LoD) 0.033 ng/mL, analytical coefficient of variation (CVA) 9%). TRAcP5b was measured using DSX^®^ TGSTA Dynex Technologies, Inc. TRAcP (Chantilly, VA, USA) (IDS-iSYS TRAcP 5b (BoneTRAP^®^), catalog no. IS-4100, Immunodiagnostic Systems Ltd., 10 Didcot Way, Boldon Business Park, Boldon, Tyne and Wear, UK) (LoD 0.9 U/L, linear range 0.9–14.0 U/L, CVA 4.5%). P1NP (IDS-iSYS Intact PINP, catalog no. IS-4000, Immunodiagnostic Systems Ltd., 10 Didcot Way, Boldon Business Park, Boldon, Tyne and Wear, UK) (LoD 2–230 ng/mL, linear range 2–230 ng/mL, CVA 5.2%) was measured using a chemiluminescence assay with TGSTA Technogenetics instrumentation (Technogenetics, Milan, Italy). All tests were performed in accordance with the manufacturer’s instructions regarding instrument and reagent handling. Multilayer internal quality control (IQC) materials provided by the manufacturer were used to verify analytical precision (CVA). Results from external quality assurance (EQA) programs (The Referenzinstitut für Bioanalytik (RfB), Friesdorfer Straße 153, Bonn, Germany) were employed to verify the accuracy. The CVA of individual analytes met the predefined objectives, and the verification of EQA exercise results showed no evidence of out-of-control analyses.

### 2.9. Statistical Analysis

To evaluate whether the number of participants necessary to ensure reliable results was appropriate, a “statistical power” using G*Power software 3.1.2 study was performed with a statistical significance target of at least 80% (*p* < 0.05) [33].

The dataset regarding the measurement of bone turnover markers (BTMs) at times t0, t1, t2, t3, and t4 was described by reporting measures of central tendency (mean and median); measures of dispersion (maximum and minimum values); measures of shape with assessment of the normality of the distribution (D’Agostino–Pearson test); and the 95% confidence interval (CI).

The “Reference Change Value (RCV),” defined as the minimum percentage change that must occur between two results of the same biomarker measured on the same subject at different times (t) to be considered statistically significant, was used to interpret the concentration variations obtained during monitoring. The RCV of the biomarkers used in the salivary matrix was 21.18%, 56.81%, and 68.44% for P1NP, TRAcP, and PTHrP, respectively (biological variability study) [34].

To compare related groups (the same biomarker measured at different measurement times), the non-parametric statistical technique of the Wilcoxon test was utilized. The test generates a *p*-value indicating the probability that the observed difference between the two compared groups of values is due to chance. If the *p*-value was lower than the significance level (*p* < 0.05), it was concluded that a significant difference exists between the two groups.

Analysis of variance (ANOVA) and ordinary least squares (OLS) multiple regression were the statistical tools used to analyze the relationships between different biomarker concentrations obtained at different times, calculating and comparing the internal variability within these groups with the variability between homogeneous groups. A high F-ratio (near 1 or greater than 1) was an indicator that the variability between group means was greater than expected by chance, leading to the rejection of the null hypothesis that all means are equal, while an F-ratio close to 1 indicated that the means were not significantly different. In particular all biomarkers showed significant deviation from normality according to the D’Agostino–Pearson test; non-parametric tests (Wilcoxon signed-rank) were used as the primary inferential method. ANOVA was applied exclusively as an exploratory tool to describe overall variability patterns across time points and was not used for confirmatory hypothesis testing.

Spearman correlation was used to visualize the relationship of the concentration of each BTM at t0, t1, t2, t3, and t4. It was accepted that an rs coefficient of +1 or −1 indicated a perfect association, while an rs of 0 (zero) would indicate the absence of association. The results were reported in tables.

To visualize data distribution and facilitate data comprehension, graphical representations were provided. Boxplots were used for the distribution of BTM values and to highlight the presence of outliers; multi-vari charts were used to visualize the percentage variations in BTMs in each patient. Statistical analysis was performed using MedCalc software, version 11.6.1.0 (MedCalc Software, Mariakerke, Belgium).

Here is the literal scientific translation of the Results, Discussion, and Conclusions sections into English. I have maintained the technical terminology and academic structure required for a scientific publication.

## 3. Results

### 3.1. Subjects Included in the Study

This study evaluated 47 subjects; the mean age was 11.3 years, and 15 subjects were female (32%). The statistical power analysis of the sample, calculated using G*Power software 3.1.2, indicated that the minimum required number was 41 with a significance level of 0.05 (*p* < 0.05). The use of RME determined in the selected patients an average increase in the intermolar width at the level of the upper first molars equal to 3.9 mm and at the level of the canines equal to 5.2 mm at t1, of 4 mm at the level of the upper first molars and of 5.4 mm at the level of the upper canines at t4.

Gingival and oral clinical conditions, as well as oral hygiene levels, were assessed at baseline and throughout the experimental period. None of the subjects presented with bleeding oral lesions of any kind; all patients maintained good oral hygiene. No significant changes were observed in PI, BOP, or PPD indices. At each orthodontic check-up, all patients had a GI < 1.

### 3.2. Salivary Measurement of BTMs

**Figure 1:**
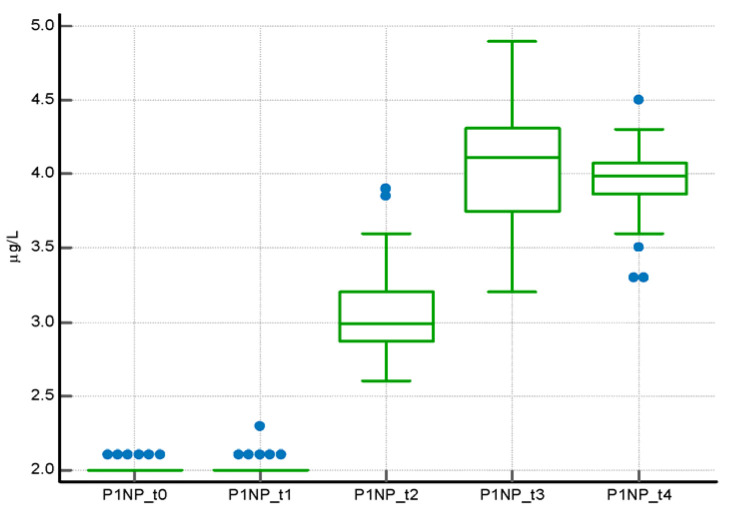

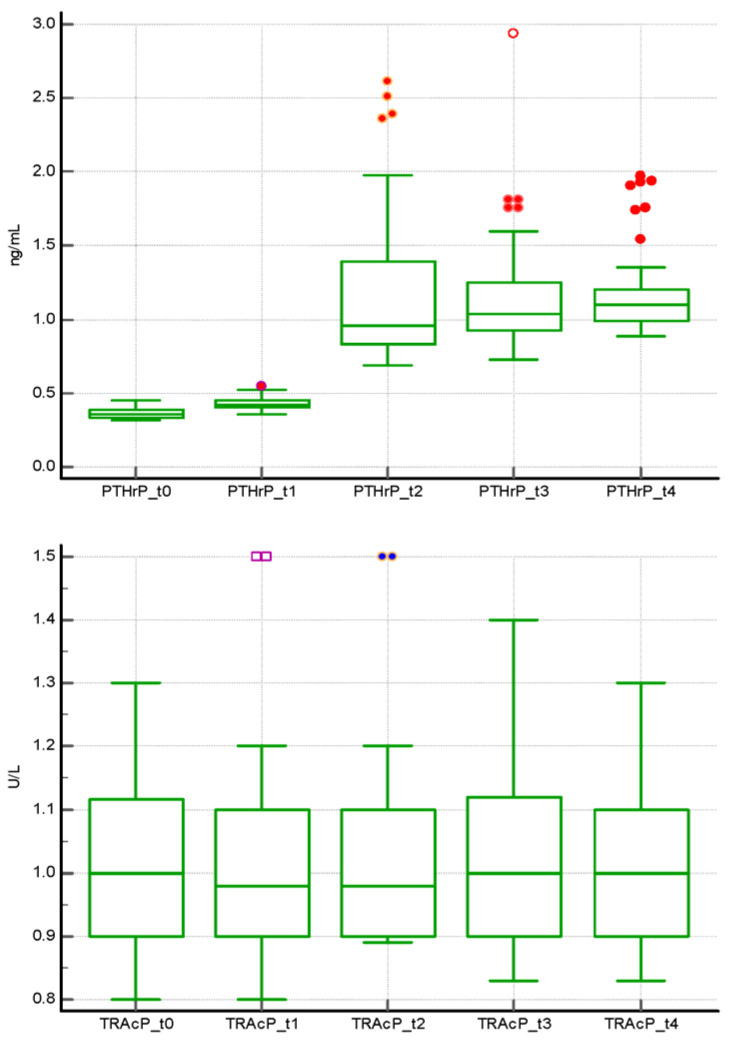
Boxplot of the concentration of PINP (µg/L), PTHrP (ng/mL) and TRAcP (U/L); the box reports the values from the 25th to the 75th quartile, the central line is the median, the horizontal lines correspond to the minimum and the maximum of the concentration; furthermore, outlier data are highlighted.

Descriptive statistics reporting the distribution of the concentrations of the different BTMs (minimum and maximum values at times t0, t1, t2, t3, and t4), mean and median values, dispersion (95% CI), and evaluation of normal distribution (D’Agostino–Pearson test) are presented in Table 1. The data show differences in minimum and maximum values, mean, and median for t0–t1 vs. t2–t3–t4 for P1NP and PTHrP. The boxplots illustrate the distribution of BTM values, showing a progressive increase from t0–t1 vs. t2–t3–t4 for P1NP and PTHrP, and the persistence of values above the LOD for TRAcP. The figures also highlight the presence of outliers for the studied BTMs with particularly high concentrations (Figure 1).

The Reference Change Value (RCV), resulting from biological and analytical variability, was used to interpret whether an observed variation during monitoring was real and not a non-significant random variation. The percentage variation between BTM concentrations at t1, t2, t3, and t4 relative to t0 was calculated; to evaluate the significance of the variation, the values obtained were compared with the “expected RCV,” obtained experimentally and reported in the literature, of 21.18%, 68.44%, and 56.81% for P1NP, PTHrP, and TRAcP, respectively (Table 2).

**Figure 2:**
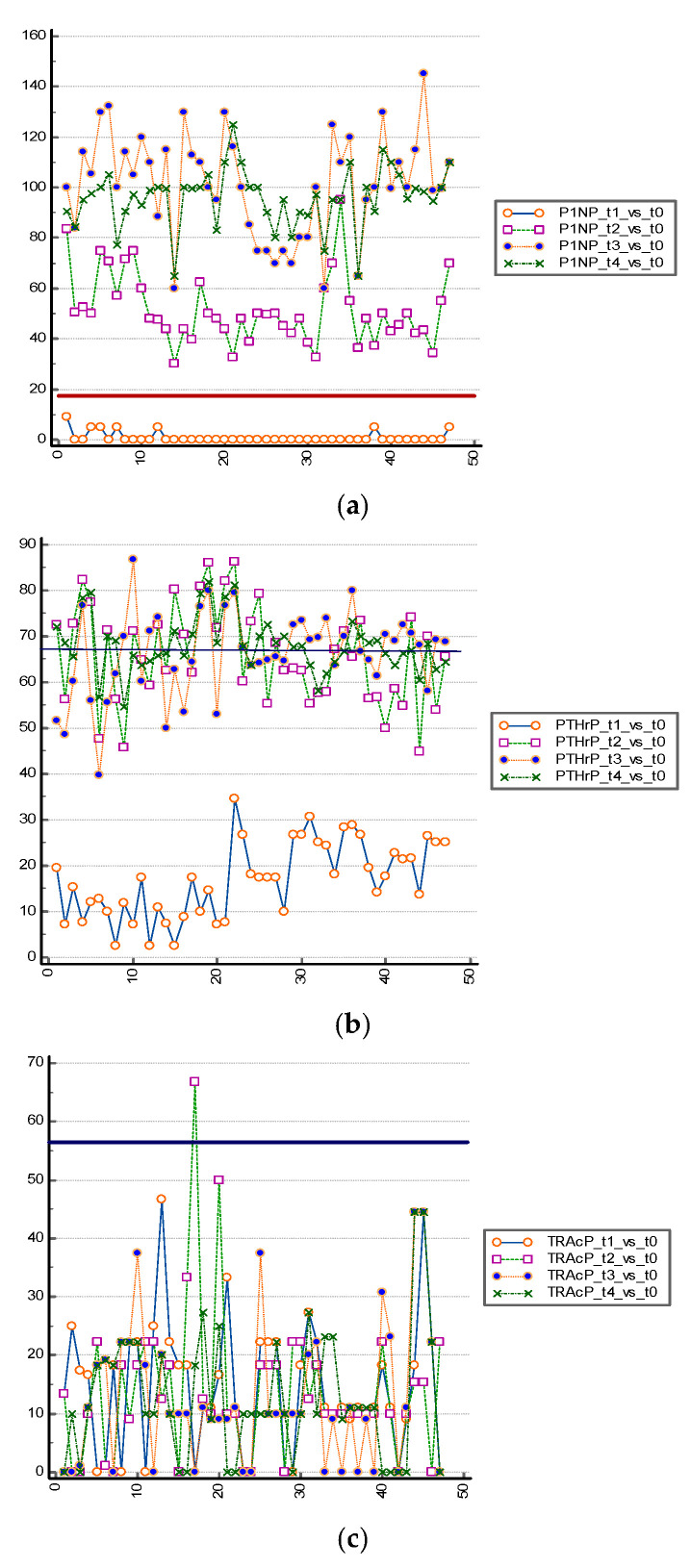
Multivariable plots of percentage change in individual patients. The percentage changes in P1NP (**a**), PTHrP (**b**), and TRAcP (**c**) concentrations at t1, t2, t3, and t4 compared to t0 are shown, assessed in each individual patient. The solid line highlights the decisional RCV for a significant change and is specific for P1NP, PTHrP, and TRAcP.

All P1NP determinations at t1,t2,t3, and t4 showed a variation greater than 21.18% (RCV), and were therefore significant compared to t0. PTHrP showed significant variations between t2 vs. t0 in 26 out of 47 subjects (55%), in 23 (48%) between t3 and t0, and in 23 (48%) between t4 vs. t0; one patient showed variations in concentration during monitoring that were not significant. For TRAcP, only one patient showed a significant variation at t2 vs. t0 (2%). The obtained data are reported in Table 2. The multivariate chart (Figure 2), showing the decision-making RCV and the percentage variations in each BTM concentration at t1,t2,t3, and t4 relative to t0 for each individual, confirmed these results.

The Wilcoxon test, a non-parametric statistical test, was used to compare related groups (the same biomarker at different measurement times). The choice of this test was based on the “non-normal” distribution of the data and the sample size. The test showed that the medians of the differences in P1NP measurements were statistically significant at all times, except between t0 and t1. Differences in PTHrP concentrations were significant except between t2 and t3 relative to t4. These results suggest that P1NP and PTHrP concentrations undergo variations with different activation and movement times; this may serve as an indicator of different phases of bone apposition or resorption. For TRAcP, no statistically significant differences were observed across all measurement times. The data are reported in Table 3.

Analysis of variance (ANOVA), used to verify whether there were significant differences between the mean concentrations of biomarkers at different times, yielded a high F-value of 3.6128 (*p* = 0.0128) for P1NP, indicating a significant difference, while the F-ratio was close to 1 for PTHrP (F = 1.3329; *p* = 0.2736) and TRAcP (F = 1.3915; *p* = 0.2534), suggesting non-significant variability between groups attributable to random variation.

**Figure 3:**
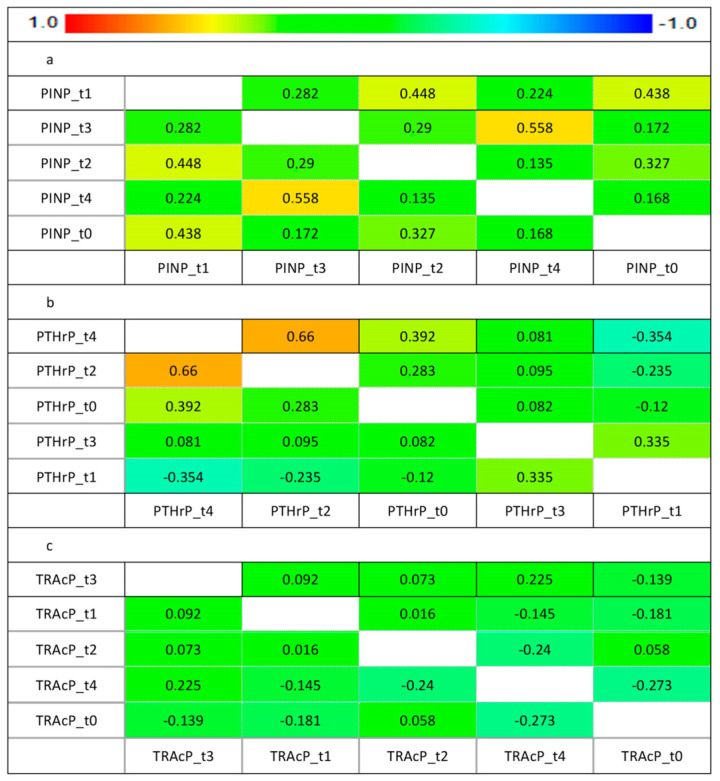
(**a**–**c**): Spearman correlation table. The rs values of P1NP (**a**), PTHrP (**b**), and TRAcP (**c**) are reported as a function of the monitoring times (t0, t1, t2, t3, t4). The colors used are indirect indicators of the rs value (1.0 = perfect positive correlation; 0 = zero correlation; −1.0 = perfect negative correlation).

The Spearman test (or Spearman correlation coefficient), a non-parametric test used to measure the strength and direction of the monotonic relationship between two variables, was used after verifying the data distribution and the monotonic relationship. P1NP showed stronger relationships between temporal variables (*rs* ranging between 0.558 and 0.135) (Figure 3), consistent with the results of other statistical tests. The applied results and statistical evaluations suggest that P1NP activity begins 30 days after the application of rapid maxillary expansion (RME), followed by a constant variation during the monitoring period (60 days). PTHrP activity occurs within the first 30 days (t2) after application, followed by no substantial variation (t3 and t4). The release process of TRAcP does not appear to produce significant variations in the evaluated time interval (t0–t4).

## 4. Discussion

The application of an RME during the developmental age involves the use of orthopedic forces ranging between 900 and 4500 g (or 9–45 newtons) for a limited period of time. These forces act primarily on the mid-palatal suture, with the goal of preserving the buccal bone of the first PERMANENT MOLARS [28,29,30]. The device features high rigidity, and the applied orthopedic forces are transmitted directly to the basal bone, producing expansion of the mid-palatal suture while reducing potential side effects (e.g., dentoalveolar tipping, bone dehiscence). The use of RME involves a separation of the articular ends of the maxillary bones, opening of the palatal suture, and an increase in palatal width [34], which is a function of the developmental stage from childhood through adolescence to adulthood.

The healing process of the achieved separation is a finely calibrated interaction between different types of bone cells, mediated by endocrine and/or paracrine signaling pathways, which are largely stimulated by biomechanical signals [35].

Following the trauma, the blood vessels within the Haversian and Volkmann canals are destroyed. This results in the depletion of oxygen and nutrient reserves, necrosis, and acidosis with the local release of growth factors [e.g., VEGF, HIF-1alpha], capable of stimulating revascularization and the maturation of stem cells (SSC) toward committed cells [36,37,38,39]. Hypoxic conditions and mechanical stimulation will determine osteoblastic or chondroblastic differentiation, giving rise to bone and cartilage, respectively.

Various biomarkers can describe the biological changes occurring during bone remodeling following orthopedic-orthodontic treatment with RME. To date, some studies have evaluated biomarkers in the gingival crevicular fluid (GCF) during rapid maxillary expansion [38]. Specifically, levels of non-specific inflammatory mediators such as interleukin-1beta, prostaglandin E2, and β-glucuronidase in the GCF recovered from the maxillary teeth of adolescents undergoing RME have increased [27,40,41,42,43]. Guerrero JA et al. [27] demonstrated in an animal model study that histological examination at the suture level, performed after RME, showed an increase in the number of osteoblasts at 7 and 14 days and an increase in key gene transcription factors (“master regulators”: Runx2, Dmp1, Col1a1), without any differentiation into osteoclasts. Perinetti et al. [44] demonstrated an increase in ALP enzymatic activity in the GCF of upper first molars during the RME retention phase (3–6 months) and in the GCF of teeth supporting the RME; they hypothesized that alveolar bone formation at tension sites may last up to 6 months of maintenance after RME.

The application of RME forces, with the opening of the mid-palatal suture, induces the stimulation of new bone apposition accompanied by remodeling and resorption phenomena with the production of bone remodeling biomarkers. The measurement of bone turnover markers (BTMs), divided into bone formation markers and bone resorption markers, can provide information on the degree of turnover. Bone formation markers primarily reflect osteoblastic bone formation activity. During bone formation, type I collagen replaces type III collagen, releasing fragments such as the type I C-terminal propeptide (P1CP) and the type I N-terminal propeptide (P1NP) [45]. P1NP reflects osteoblast activity, is a more sensitive bone formation marker than others, and its levels are equimolar to the collagen incorporated into the bone matrix and significantly correlated with histomorphometry and bone formation measurements [46,47,48,49].

TRAcP-5b, a 35–37 kDa glycoprotein and acid phosphatase (ACP) isoenzyme, is produced by osteoclasts and released into bone resorption lacunae [47]. It plays a role in the degradation of type I collagen in the bone matrix; its concentration reflects bone resorption and promotes osteoclast migration. TRAcP-5b concentrations reflect the number of osteoclasts rather than their activity. PTHrP is involved in regulating bone remodeling activities through autocrine and paracrine hormonal actions. PTHrP, produced by dental pulp cells, coordinates cellular activity in resorption and apposition processes during tooth eruption; it acts on osteoclasts for necessary resorption of the bone overlying the crown and on osteoblasts to form bone at the base of the tooth, pushing it toward the top of the crypt [48].

This is the first study to evaluate the dosage of P1NP, TRAcP, and PTHrP in the saliva of patients undergoing RME treatment. It was considered that the assessment of the dynamic process of bone remodeling during RME did not lend itself to interpretation based on the measurement of a single bone biomarker; therefore, variations in a bone deposition biomarker (P1NP), a resorption biomarker (TRAcP), and a regulatory biomarker (PTHrP) were monitored [48]. The selection of P1NP, TRAcP, and PTHrP took into account the limits of detection and quantification of the methods in use and the results of previous studies on the use of bone metabolism biomarkers in the salivary matrix.

The results obtained demonstrated a statistically significant difference for P1NP between the 30th (t2) and 45th (t3) days of therapy, with a peak in deposition. This suggests that the initial phase of bone deposition begins between day 30 and 45. Conversely, TRAcP showed no statistically significant variation, and PTHrP changed significantly during the first 30 days, while it underwent no significant variations in the subsequent period. The lack of a consistent, time-dependent pattern in PTHrP levels, together with the marked inter-individual variability, supports the interpretation that this molecule is released through paracrine mechanisms aimed at local tissue regulation rather than exerting a direct systemic effect on bone.

This study involved the collection of five saliva samples at specific times: before the start of orthodontic treatment (t0), and subsequently at 15 days (t1), 30 days (t2), 45 days (t3), and 60 days (t4) from the start of treatment—a duration considered adequate to allow for the activation of remodeling processes and thus for monitoring. The results suggested that it is possible to obtain information on the state of bone remodeling in an individual subject, especially if monitoring is performed for a sufficient time to allow for the activation of remodeling processes. The behavior of the biomarkers studied in the first 60 days after RME application suggests that, in this phase, stimuli for bone deposition prevail over resorption stimuli, both at the level of the palatal suture and at the periodontal level on the RME anchorage teeth. The median concentration of P1NP in the patients undergoing RME evaluated by us was 4.11 μg/L (CI 3.99 to 4.20) at t3 and 3.99 μg/L (CI 3.92 to 4.00) at t4; the median concentration of PTHrP was 0.96 ng/mL (CI 0.89 to 1.23) at t2 and 1.04 ng/mL (CI 0.97 to 1.18) at t3.

All subjects achieved the expected clinical effect of rapid maxillary expansion; however, only salivary biomarkers allowed us to describe the temporal, non-clinically visible, progression of the underlying biological remodeling.

In our study, the temporal behavior of the biomarkers was interpreted in relation to the biomechanics of rapid maxillary expansion. The progressive increase in P1NP observed between t2 and t4 is consistent with the biological sequence triggered by RME: an initial phase (t0–t1) mainly characterized by mechanical separation of the mid-palatal suture, followed by a phase of collagen deposition and new bone formation (t2–t4), during which osteoblastic activity increases in response to orthopedic loading. This pattern aligns with previous evidence describing osteogenic activation after mechanical stimulation.

Conversely, the absence of significant changes in TRAcP suggests that osteoclastic activity may be less prominent during the early weeks of expansion, with bone deposition processes prevailing over resorption. It is also possible that TRAcP exhibits lower sensitivity in the salivary matrix compared with other biological fluids, limiting the detection of subtle variations in osteoclastic activity.

The variability observed for PTHrP may reflect the complex and context-dependent regulatory role of this peptide in bone remodeling. PTHrP is known to exhibit a biphasic and non-linear behavior, modulated by the local microenvironment and mechanical stress. The fluctuations detected in our study may therefore represent local regulatory adjustments occurring during the early phases of sutural adaptation. However, given the preliminary nature of this investigation, further studies are needed to better characterize the physiological significance of this biomarker in the context of RME.

Overall, integrating the temporal trends of the biomarkers with the physiology of bone remodeling suggests that the biochemical response to RME during the first weeks is dominated by bone formation processes, whereas the contribution of bone resorption appears less evident in saliva.

Beyond the descriptive interpretation of biomarker trends, it is important to critically evaluate the biological plausibility of these findings in light of existing evidence. The progressive increase in P1NP between t2 and t4 is consistent with the osteogenic response described in histological studies of mid-palatal suture adaptation, where early mechanical separation is followed by collagen deposition and new bone formation [16,18]. This interpretation is further supported by reports of increased osteoblast number and elevated expression of alkaline phosphatase and type I collagen following RME-induced mechanical loading [23].

The absence of significant TRAcP variation may indicate that early osteoclastic activity is limited during the initial phases of expansion, or that salivary TRAcP is less sensitive than other matrices for detecting subtle changes in bone resorption. This is consistent with previous studies showing that salivary biomarkers may exhibit lower responsiveness compared with gingival crevicular fluid, particularly for resorption-related markers [27].

The variability observed in PTHrP levels may reflect the complex regulatory behavior of this peptide, which is known to exhibit context-dependent and non-linear dynamics during skeletal adaptation. Inter-individual differences in sutural maturation and functional loading—well documented in anatomical and biomechanical studies [19]—may also contribute to the heterogeneous PTHrP response observed in our cohort.

The study of variations in biomarkers related to bone turnover for understanding bone remodeling processes may introduce new opportunities in orthodontics. Integration with further information on the process occurring in periodontal tissues during orthopedic–orthodontic therapies would make the selection of mechanical load and treatment duration more effective, reducing the likelihood of associated adverse events. Current approaches for assessing and monitoring RME rely on clinical evaluation and on intraoral scanning or Cone Beam Computed Tomography (CBCT) [9]. However, these techniques are unable to detect very small dental displacements and do not provide sufficient insight into the biological events occurring in the alveolar bone and periodontal ligament during treatment. As reported in the literature, the use of scanners presents several limitations, including dependence on the operator’s experience and manual skills, the presence of blood or saliva during impression taking, the need for rescanning, and the variability introduced during post-processing procedures [48,49].

CBCT, on the other hand, is a radiographic method that, although low-dose compared to conventional medical CT, still exposes the patient to radiation; it offers static images of the bone structure with morphological and structural data (density, thickness, dimension) without explaining the ongoing metabolic dynamics [50].

Monitoring salivary concentrations of bone metabolism biomarkers, evaluated by comparing the percentage deviation with the RCV [34] could be used during RME treatment to determine the amount and duration of the force to be applied. Saliva sample collection is a non-invasive procedure that does not expose the patient to any biological risk, provides a dynamic view of the metabolic imbalance occurring at the start of treatment, is an objective and quantifiable measure (expressed in μg/L or ng/mL), and is not influenced by the experience or manual skills of the operator collecting the sample. Therefore, biomarkers in saliva lend themselves to being used to optimize orthopedic–orthodontic treatment in the individual subject. Future studies combining biomarker analysis, imaging, and clinical parameters will be essential to fully elucidate the mechanistic sequence of sutural response to rapid maxillary expansion.

## 5. Conclusions

This study provided biochemical evidence of the bone remodeling processes that take place during RME. Monitoring with serial sampling over a total period of 60 days highlighted that the biomarkers descriptive of apposition phenomena vary significantly compared to those of resorption. Subsequent studies involving longer monitoring times may clarify whether patients with particularly significant deviations in biomarker concentrations may present a higher risk of potential late complications from RME orthodontic therapy.

## Figures and Tables

**Table 1:** Descriptive statistical analysis of PINP, PTHrP and TRAcP concentrations on salivary matrix, stratified over time (t0, t1, t2, t3, and t4) (CI = confidence interval).

	P1NP (µg/L) t0	P1NP (µg/L) t1	P1NP (µg/L) t2	P1NP (µg/L) t3	P1NP (µg/L) t4	PTHrP (ng/mL) t0	PTHrP (ng/mL) t1	PTHrP (ng/mL) t2	PTHrP (ng/mL) t3	PTHrP (ng/mL) t4	TRAcP (U/L) t0	TRAcP (U/L) t1	TRAcP (U/L) t2	TRAcP (U/L) t3	TRAcP (U/L) t4
N.	47	47	47	47	47	47	47	47	47	47	47	47	47	47	47
Minimum	2.00	2.00	2.60	3.20	3.30	0.32	0.36	0.69	0.73	0.89	0.8	0.8	0.89	0.83	0.83
Maximum	2.10	2.29	3.90	4.90	4.50	0.45	0.55	2.61	2.94	1.97	1.3	1.5	1.5	1.4	1.3
Mean	2.01	2.02	3.05	4.05	3.94	0.36	0.43	1.02	1.16	1.18	1.03	1.02	1.03	1.03	1.01
95% CI	2.00 to 2.02	2.00 to 2.02	2.96 to 3.13	3.93 to 4.18	3.87 to 4.01	0.35 to 0.37	0.42 to 0.44	1.05 to 1.34	1.04 to 1.27	1.09 to 1.26	0.99 to 1.07	0.97 to 1.06	0.98 to 1.07	0.99 to 1.07	0.98 to 1.05
Median	2.00	2.00	2.99	4.11	3.99	0.3 6	0.42	0.96	1.04	1.10	1.00	0.98	0.98	1.00	1.00
95% CI	2.00 to 2.00	2.00 to 2.00	2.90 to 3.00	3.99 to 4.20	3.92 to 4.00	0.34 to 0.37	0.40 to 0.45	0.89 to 1.23	0.97 to 1.18	1.01 to 1.13	0.97 to 1.10	0.90 to 1.10	0.90 to 1.10	0.925 to 1.10	0.90 to 1.00
Normal Distr.	<0.0001	<0.0001	0.0014	0.0056	0.008	0.0003	0.0019	<0.0001	<0.0001	<0.0001	0.022	<0.0001	<0.0001	0.0006	0.0001

**Table 2:** Percentage change in P1NP, PTHrP, and TRAcP concentrations at t1, t2, t3, and t4 compared to t0. The table shows in gray the “non-significant” changes obtained by comparison with the “expected RCV” (21.18%, 68.44%, and 56.81% for P1NP, PTHrP, and TRAcP, respectively).

Patient	P1NP t1 vs. t0	P1NP t2 vs. t0	P1NP t3 vs. t0	P1NP t4 vs. t0	PTHrP t1 vs. t0	PTHrP t2 vs. t0	PTHrP t3 vs. t0	PTHrP t4 vs. t0	TRAcP t1 vs. t0	TRAcP t2 vs. t0	TRAcP t3 vs. t0	TRAcP t4 vs. t0
1	9.05	83.33	100.00	90.48	19.44	72.61	51.69	72.08	13.33	13.33	0.00	0.00
2	0.00	50.50	84.00	84.50	7.14	56.18	48.68	68.50	25.00	0.00	0.00	10.00
3	0.00	52.38	114.29	95.24	15.38	72.73	60.24	65.63	17.43	0.00	1.12	0.00
4	5.00	50.00	105.50	97.50	7.69	82.43	76.80	78.35	16.67	10.00	11.11	11.11
5	5.00	75.00	130.00	100.00	12.20	77.50	56.10	79.55	0.00	22.22	18.18	18.18
6	0.00	70.50	132.50	105.00	12.82	47.62	39.73	56.86	1.11	1.12	19.09	19.09
7	4.76	57.14	100.00	77.14	10.00	71.43	55.56	70.00	18.18	0.00	0.00	18.18
8	0.00	71.43	114.29	90.48	2.50	56.18	61.76	69.09	0.00	18.18	22.22	22.22
9	0.00	75.00	105.00	97.00	11.76	45.78	69.91	54.55	22.22	9.09	22.22	22.22
10	0.00	60.00	120.00	93.00	7.14	71.11	86.73	65.79	22.22	18.18	37.50	22.22
11	0.00	48.00	110.00	99.00	17.50	64.89	60.24	63.74	0.00	22.22	18.18	10.00
12	4.76	47.62	88.57	100.00	2.50	59.38	71.11	64.55	25.00	22.22	0.00	10.00
13	0.00	44.00	115.00	99.50	10.87	72.67	74.21	65.83	46.67	12.50	20.00	20.00
14	0.00	30.00	60.00	65.00	7.50	62.63	50.00	66.36	22.22	18.18	10.00	10.00
15	0.00	44.00	130.00	100.00	2.50	80.30	62.86	71.11	18.18	0.00	10.00	0.00
16	0.00	39.50	113.00	99.50	8.89	70.50	53.41	65.83	18.16	33.33	10.00	0.00
17	0.00	62.50	110.00	100.00	17.50	62.07	64.52	70.54	0.00	66.67	0.00	18.18
18	0.00	50.00	100.00	105.00	10.00	80.95	76.62	79.31	11.11	12.50	11.11	27.27
19	0.00	48.00	95.00	83.00	14.63	86.06	80.11	81.87	11.11	10.00	9.09	9.09
20	0.00	44.00	130.00	110.00	7.14	71.94	53.01	68.55	16.67	50.00	9.09	25.00
21	0.00	32.50	116.00	125.00	7.69	82.20	76.80	78.68	33.33	10.00	9.09	0.00
22	0.00	48.00	100.00	110.00	34.55	86.21	79.55	81.15	11.11	10.00	11.11	0.00
23	0.00	39.00	85.00	100.00	26.67	60.24	67.65	68.81	0.00	0.00	0.00	10.00
24	0.00	50.00	75.00	100.00	18.18	73.33	63.64	63.64	0.00	0.00	0.00	10.00
25	0.00	49.50	75.00	90.00	17.50	79.25	64.13	70.00	22.22	18.18	37.50	10.00
26	0.00	50.00	70.00	80.00	17.50	55.41	64.89	72.50	22.22	18.18	10.00	10.00
27	0.00	45.00	75.00	95.00	17.50	68.57	65.63	68.65	22.22	18.18	10.00	22.22
28	0.00	42.00	70.00	80.00	10.00	62.50	64.71	69.97	0.00	0.00	10.00	10.00
29	0.00	48.00	80.00	90.00	26.67	62.92	72.50	67.65	0.00	22.22	10.00	0.00
30	0.00	38.50	80.00	89.00	26.67	62.50	73.60	68.00	18.18	22.22	10.00	10.00
31	0.00	32.50	100.00	97.00	30.61	55.26	69.37	63.83	27.27	12.50	20.00	27.27
32	0.00	60.00	60.00	75.00	25.00	57.61	69.77	58.06	22.22	18.18	22.22	10.00
33	0.00	70.00	125.00	95.00	24.44	58.02	73.85	61.80	11.11	10.00	0.00	23.08
34	0.00	95.00	110.00	95.00	18.18	67.27	63.64	68.96	9.09	10.00	9.09	23.08
35	0.00	55.00	120.00	110.00	28.26	71.05	70.00	66.67	11.11	10.00	0.00	9.09
36	0.00	36.50	65.00	65.00	28.89	65.59	80.00	73.33	9.09	10.00	11.11	11.11
37	0.00	48.00	95.00	100.00	26.67	73.39	66.67	70.00	11.11	10.00	0.00	11.11
38	4.76	37.14	100.00	90.48	19.51	56.58	64.89	68.50	9.09	10.00	9.09	11.11
39	0.00	50.00	130.00	115.00	14.29	56.63	61.29	69.04	11.11	10.00	0.00	11.11
40	0.00	43.00	99.50	110.00	17.78	50.00	70.40	66.36	18.18	22.22	30.77	0.00
41	0.00	45.50	110.00	105.00	22.73	58.54	69.09	63.83	11.11	10.00	23.08	0.00
42	0.00	50.00	100.00	95.50	21.43	54.79	72.50	66.33	0.00	0.00	0.00	0.00
43	0.00	42.00	115.00	99.50	21.74	74.29	70.73	66.97	9.09	10.00	11.11	0.00
44	0.00	43.50	145.00	98.50	13.64	44.93	68.87	60.42	18.18	15.38	44.44	44.44
45	0.00	34.50	99.00	94.50	26.53	70.00	58.14	68.27	44.44	15.38	44.44	44.44
46	0.00	55.00	99.50	100.00	25.00	53.85	69.38	62.89	22.22	0.00	22.22	22.22
47	5.00	70.00	110.00	110.00	25.00	65.63	68.87	64.52	0.00	22.22	0.00	0.00

**Table 3:** Comparison of the median differences in P1NP, PTHrP, and TRAcP measurements at different sampling times (t0, t1, t2, t3, and t4) (Wilcoxon test). Statistically significant differences (*p* < 0.05) are highlighted in blue.

Sample	Two-Tailed Probability	Sample	Two-Tailed Probability	Sample	Two-Tailed Probability
P1NP t0 vs. P1NP t1	*p* = 0.5781	PTHrP t0 vs. PTHrP t1	*p* < 0.0001	TRAcP t0 vs. TRAcP t1	*p* = 0.3574
P1NP t0 vs. P1NP t2	*p* < 0.0001	PTHrP t0 vs. PTHrP t2	*p* < 0.0001	TRAcP t0 vs. TRAcP t2	*p* = 0.2610
P1NP t0 vs. P1NP t3	*p* < 0.0001	PTHrP t0 vs. PTHrP t3	*p* < 0.0001	TRAcP t0 vs. TRAcP t3	*p* = 1.0000
P1NP t0 vs. P1NP t4	*p* < 0.0001	PTHrP t0 vs. PTHrP t4	*p* < 0.0001	TRAcP t0 vs. TRAcP t4	*p* = 0.8763
P1NP t1 vs. P1NP t2	*p* < 0.0001	PTHrP t1 vs. PTHrP t2	*p* < 0.0001	TRAcP t1 vs. TRAcP t2	*p* = 0.7096
P1NP t1 vs. P1NP t3	*p* < 0.0001	PTHrP t1 vs. PTHrP t3	*p* < 0.0001	TRAcP t1 vs. TRAcP t3	*p* = 0.1260
P1NP t1 vs. P1NP t4	*p* < 0.0001	PTHrP t1 vs. PTHrP t4	*p* < 0.0001	TRAcP t1 vs. TRAcP t4	*p* = 0.2725
P1NP t2 vs. P1NP t3	*p* < 0.0001	PTHrP t2 vs. PTHrP t3	*p* < 0.0001	TRAcP t2 vs. TRAcP t3	*p* = 0.1277
P1NP t2 vs. P1NP t4	*p* < 0.0001	PTHrP t2 vs. PTHrP t4	*p* = 0.9705	TRAcP t2 vs. TRAcP t4	*p* = 0.1652
P1NP t3 vs. P1NP t4	*p* < 0.0001	PTHrP t3 vs. PTHrP t4	*p* = 0.4157	TRAcP t3 vs. TRAcP t4	*p* = 0.5888

## Data Availability

The data presented in this study are available upon request from the corresponding authors due to privacy reasons (sensitive data).
